# Supplementary figures and images for: Interplay between axonal Wnt5-Vang and dendritic Wnt5-Drl/Ryk signaling controls glomerular patterning in the Drosophila antennal lobe
Source: PLoS Genet. 2020 May 1;16(5):e1008767. doi: 10.1371/journal.pgen.1008767 (PMC7219789; doi:10.1371/journal.pgen.1008767)

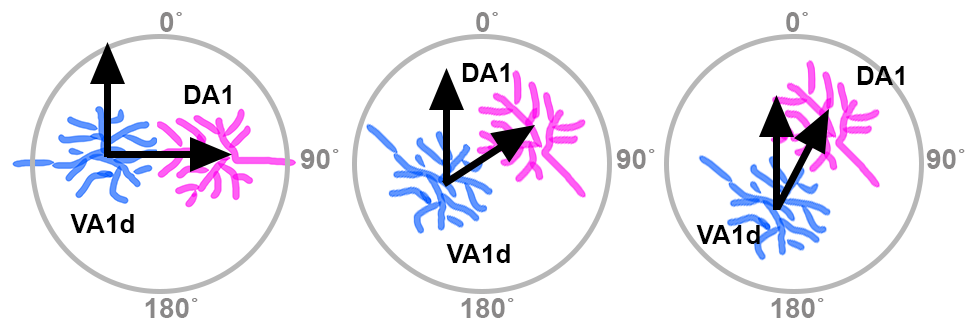

Supplement: S1 Fig — Schematic representations of the frontal views of left ALs are shown (dorsal/0˚ is up and lateral/90˚ is to the right). A line was drawn through the centers of the DA1 and VA1d glomeruli. Where the line intersects with the dorsal-ventral axis, the angle in the clockwise direction from 0˚ was measured. During development, the DA1/VA1d angle decreases because the two glomeruli rotate around each other in the counterclockwise direction. (TIF) [file pgen.1008767.s001.tif]

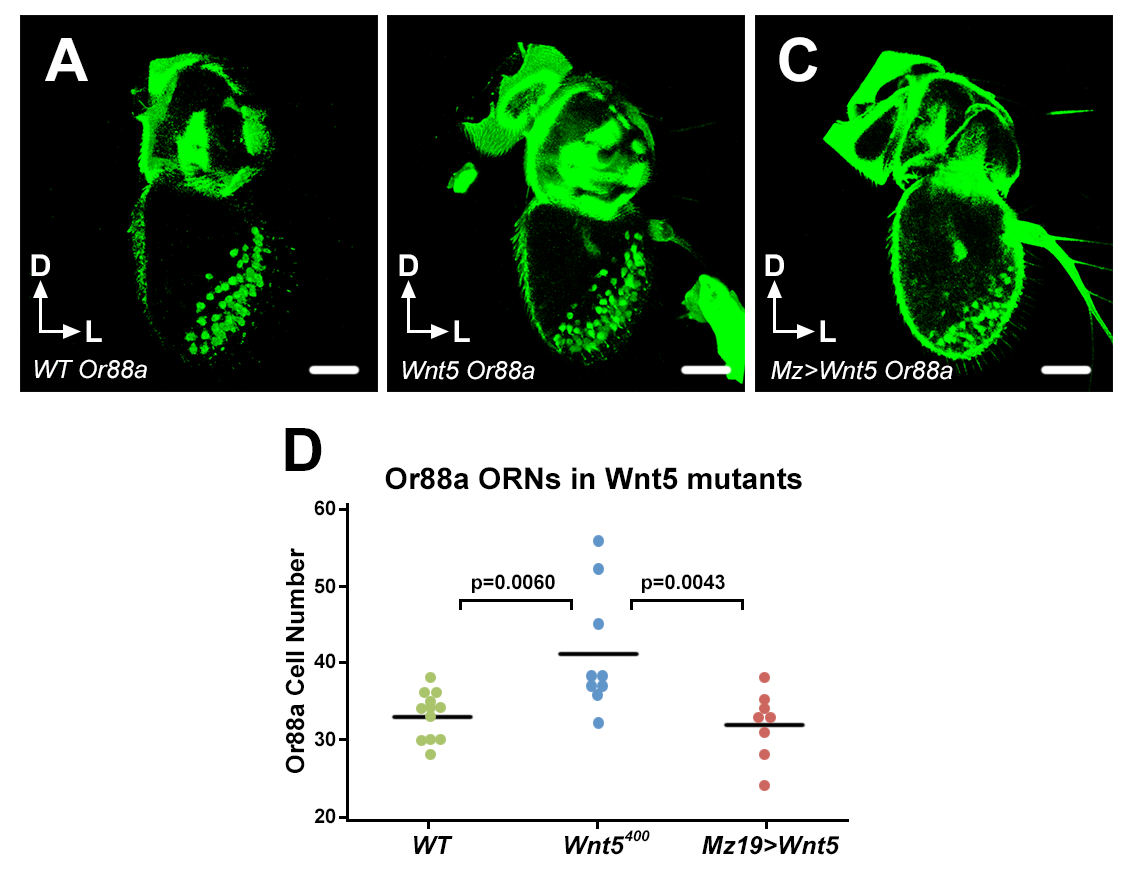

Supplement: S2 Fig — Frontal views of adult left antennae are shown (dorsal up and lateral to the right. (A-C) Representative freshly dissected antennae from animals expressing Or88a-mGFP were imaged using the confocal microscope to visualize the live Or88a neurons in the wild-type (A), Wnt5400 (B), and Mz19-Gal4 UAS-Wnt5 (C) animals. (D) Quantification of the Or88a neuronal numbers in the different genotypes. The numbers of Or88a neurons in the Wnt5400 (41.22 ± 2.681, N = 9) mutant and Mz19-Gal4 UAS-Wnt5 (32.00 ± 1.535, N = 8) overexpression animals are similar to those in the Wild type (33.17 ± 0.8776, N = 12). Wild type vs Wnt5400, p = 0.0060; Wild type vs Mz19-Gal4 UAS-Wnt5, p = 0.8834; Wnt5400 vs Mz19-Gal4 UAS-Wnt5, p = 0.0043; one-way ANOVA with post hoc Tukey test. (TIF) [file pgen.1008767.s002.tif]
